# Factor structures of the Swedish Version of the RFIPC: Investigating the Validity of Measurements of IBD Patients’ Worries and Concerns

**DOI:** 10.4021/gr247w

**Published:** 2010-09-20

**Authors:** Susanna Jaghult, Fredrik Saboonchi, Unn-Britt Johansson, Regina Wredling, Marjo Kapraali

**Affiliations:** a Karolinska Institutet, Department of Clinical Sciences, Danderyd Hospital, Division of Medicine, SE-182 88 Stockholm, Sweden; b Sophiahemmet University College, Box 5605, SE-114 86 Stockholm, Sweden

**Keywords:** Concern, Crohn’s disease, Health-related quality of life, Inflammatory bowel disease, Ulcerative colitis, Worry

## Abstract

**Background:**

Worries and concerns of patients with IBD comprise an important negative factor in their HRQOL. The Rating Form of Inflammatory Bowel Disease Patient Concerns (RFIPC) was developed to describe the nature and degree of the worries and concerns of IBD patients. In the original version, the specific issues of worries are divided into four separate factors. These factors provide useful information about HRQOL and the kind of worries and concerns which are most important to the patient. However, the Swedish version of the RFIPC is often scored using a single sum score, implying that all the specific issues of worries stem from a single general worry factor. The aim of this study was to validate the factor structure of the Swedish version of the RFIPC.

**Methods:**

A sample consisting of 195 patients with IBD filled out the RFIPC. Confirmatory factor analysis was performed to examine fit of three hypothesized models of factor structure. Spearman’s correlation and Mann-Whitney analysis were used to follow up the results.

**Results:**

The single-factor model displayed poor fit indices. The four-factor model marked substantive improvement, but still remains inadequate. The final four-factor model permitting correlated error terms between some items displayed the most adequate fit.

**Conclusions:**

The factorial structure of the RFIPC, as suggested in the original version, was able to be replicated with a slight modification in the Swedish version. The separate factors identified in this structure provide more detailed information about the worries and concerns of IBD patients as these components of worries are different related to HRQOL and general health.

## Introduction

The Rating Form of Inflammatory Bowel Disease Patient Concerns (RFIPC) was developed by Drossman et al to describe the nature and degree of the worries and concerns expressed by people with inflammatory bowel diseases [1-2]. The Swedish version of this questionnaire has been evaluated among Swedish patients [3] and is used extensively [4-9]. However, the scoring procedure for the Swedish version of the RFIPC is most often performed without using the factor structure suggested in the original version. The different approaches to the scoring procedures may have direct implications for the measurement of patients’ worries and concerns in clinical care.

Inflammatory bowel diseases (IBD), including Crohn’s disease (CD) and ulcerative colitis (UC), are chronic diseases, characterized by alternating periods of remission with relapses [10]. IBD is a lifelong illness that can impact patients’ daily lives as well as their attitudes, fears, and beliefs [11]. Worries and concerns related to IBD and its consequences may affect the patient’s adjustment to illness, and satisfaction and compliance with treatment [1-2].

Studies have shown that patients with IBD rate their health-related quality of life (HRQOL) lower, as compared with a general population [7, 12-18]. HRQOL is determined by the patient’s physical, psychological and social status, as well as attitudes, concerns and behaviours in response to the disease [2]. Living with a chronic disease involves uncertainty about the extent and treatment of the disease, and this can also result in a sense of powerlessness – a feeling of losing control over one’s life due to the disease [19]. This sense of uncertainty may also be reflected in the patient’s worries and concerns [1].

There is evidence that psychological factors play an independent role in the course of IBD. Psychological distress is frequent in IBD and psychological factors are related to exacerbation of and coping with IBD [20-23]. Psychological factors such as depressive mood associated with anxiety and impaired HRQOL after a relapse may influence the further course of IBD. Depressive mood represents a further risk factor for clinical recurrence of disease that should warrant more consideration in the clinical treatment of patients with IBD [20]. Patients with IBD express a frequent need for psychological interventions that is much higher than in rheumatoid arthritis [24]. It is recommended that physicians routinely screen their IBD patients for anxiety and depressive disorders in the regular course of providing care, especially at the time of first diagnosis and during disease flares [20, 25]. Effective treatment of anxiety or depression can decrease suffering and lead to improved quality of life [25]. Physicians should also be attentive to the worries and concerns patients have about their illness. Depressive coping may also influence non-specific somatic complaints, psychological distress, and IBD-related concerns [26].

The RFIPC has been shown to be a reliable and valid measure of the worries and concerns expressed by patients with IBD, for use in clinical care and research [1]. It has for example been used to characterize HRQOL among patients with IBD [2, 5, 7-9, 27-29], to identify the most important worries and concerns in IBD [4, 26, 30-33], to make comparisons between IBD and other chronic diseases [24] and to identify different cross-cultural patterns in RFIPC results [7, 34].

Development of the RFIPC, a factor analysis of the 25 included items in the scale, yielded four factors that explained 88% of the total variance of the scale. The factors are: impact of the disease, sexual intimacy, complications of the disease and body stigma [1]. The four factors provide useful information about HRQOL and the kind of worries and concerns which are most important to the patient [2, 24, 26-28, 31, 34]. This information can help physicians and nurses to provide more specific education and support by focusing on the most pronounced dimension of patients’ worries. For example, in one study the aim was to examine the impact of perceived body stigma on IBD patients, and therefore the factor body stigma was the only factor that was investigated [35].

However, instead of using these four factors, it is mostly the sum score that is reported in the studies using the Swedish version [3-9, 36]. The sum score (one single-factor) of the RFIPC has been used for comparison between different diagnoses, different sexes, relapse versus remission, or different countries [7-8, 32]. Implicitly, this approach considers the specific worries and concerns to be manifestations of a general underlying dimension of worries. To our knowledge, the factor structure of the RFIPC in Sweden has not yet been subjected to empirical investigation. An exception is the study by Bergquist et al, in which an exploratory factor analysis was unable to distinguish any factors in the RFIPC items [36]. This study did not provide a detailed report of the factor analysis underlying this conclusion. An exploratory factor analysis is primarily suitable at the development stage of a measurement [37]. When a theory or a previously established structure is accessible, a confirmatory approach is recommended [37].

If the patients’ worries and concerns can be considered to consist of distinct components, rather than a single general dimension, the RFIPC provides much more specific, more detailed and valuable information about the patients’ HRQOL. This possibility can be addressed by examining the factorial structure of the RFIPC.

The aim of this study is to validate the factor structure in the Swedish version of the RFIPC and to examine how these hypothesized different factors are associated with HRQOL and general health.

## Patients and Methods

### Patients

A sample consisting of 195 patients filled out the RFIPC. The patients included in the study had a confirmed diagnosis of CD or UC and were all receiving care at the IBD clinic at Danderyd Hospital, Stockholm. The inclusion criteria were: being in clinical remission, having no other chronic disease, having a good understanding of the Swedish language, and being able to fill in a questionnaire. Clinical remission was defined as having no bowel symptoms associated with active disease, i.e., no diarrhoea or blood in stools, and receiving no acute treatment. The patients that fulfilled the inclusion criteria were sent a letter including written information about the study, one questionnaire with demographic data, the RFIPC and two other questionnaires that measured HRQOL and general health (all described below).

### Instruments

The RFIPC consists of 25 items of concern related to IBD. Each item is graded on a 100 mm visual analogue scale, where the extremes are 0 mm = “not at all” and 100 mm = “a great deal”. The basic formulation is “Because of your condition, how concerned are you with…?” The items are, for example, “having surgery” and “feeling alone”. In the original version, a mean is reported for each item, as well as the sum score, which is the mean of the 25 items [1], while in the Swedish version a median is most often used [3-5, 7-8].

The Health Index (HI) questionnaire contains nine questions that describe patients’ general health [38]. Each question is graded: 1 = very poor, 2 = rather poor, 3 = rather good, 4 = very good. The total score ranges from 9 (very poor health) to 36 (very good health). The questionnaire includes questions regarding energy, temper, fatigue, loneliness, sleep, vertigo, bowel function, pain and mobility. The internal consistency, Cronbach’s alpha coefficient, in the present study was 0.82.

The Inflammatory Bowel Disease Questionnaire (IBDQ) is used to assess HRQOL for patients with IBD. The questionnaire has 32 items, divided into four subscales, assessing bowel symptoms (bowel movements and abdominal pain), systemic symptoms (fatigue and sleep), emotional function (irritation, depression, and aggression) and social function (ability to work and participate in social activities). The questionnaire has been shown to be a reliable and sensitive measure of HRQOL [39] and has been validated in Sweden [40-41]. In this study the response option that is used in the UK version of the IBDQ is used, since it is more differentiated[42]. In this version, a 4-graded Likert scale is used instead of the 7-graded Likert scale that was developed by Guyatt et al [43]. Score 1 represents the “best function” and score 4 represents the “worst function”. All 32 items are used [43] and the total score ranges from 32 (optimal HRQOL) to 128 (worst HRQOL). The modified version of the IBDQ was tested for reliability and validity in a previous study [44]. The internal consistency, Cronbach’s alpha coefficient, in the present study was 0.93.

### Statistical analysis

Basic descriptive statistics concerning illness-related factors, demographic variables and RFIPC scores were conducted using the statistical program Statistical Package for Social Sciences software (SPSS) 17.0 for Windows.

Confirmatory factor analysis (CFA) was performed on the variance-covariance matrix of the RFIPC items, using the Mplus for Windows Version 5 and AMOS Version 17. CFA was performed using the MLR estimator for achieving robust standards error and test statistics due to concerns about departure from normality. The fit of CFA models was assessed using the robust Yuan-Bentler T2* Chi-square test statistics and comparable fit index (CFI) [45]. Badness of fit was assessed by the standardized root mean squared residual (SRMR) and the root mean squared error of approximation (RMSEA) [46]. For the SRMR a cut-off value close to 0.08 or below is recommended, and for the RMSEA a cut-off of < 0.06 is recommended. A combination of the SRMR and RMSEA minimizes the rejection of well-fitting models [46].

Cronbach’s alpha coefficient was obtained to test reliability in terms of internal consistency. Spearman’s correlation analysis was used to follow up the results from CFA. The Bonferroni-adjusted significance level at 0.01 was used because multiple correlations were examined. In addition, Mann-Whitney U test was also performed to follow up the results from CFA.

### Models of the RFIPC included in the analysis

The first model to be validated was the single-factor model of the RFIPC. This model is based on the assumption that the variance in the RFIPC can be partitioned into one single factor of worry plus error variance associated with each individual item [37]. Testing the fit of a one-factor model corresponds both with the statistical evaluation of the most parsimonious of all the possible models [37], and also with an often used scoring procedure of the RFIPC in Sweden.

The second model in the analysis was the four-factor model according to Drossman et al (1991), reporting factor structure based on an exploratory factor analysis using maximum likelihood method with varimax rotation [1]. In this model the RFIPC consists of four distinct factors: impact of disease, sexual intimacy, complications, and body stigma.

The third and final model in the analysis was the above-mentioned four-factor model according to Drossman et al, with the addition of correlated error terms between item 5 (developing cancer) and item 6 (dying early), and item16 (having surgery) and item 17 (having an ostomy bag). Although it is generally recommended not to include correlated error terms for subgroups of items, the specification of these correlated error terms was justified by clinical observations. For example, having an ostomy bag is often a consequence of surgery, and the fear of dying early is normally related to concerns about developing cancer. The correlation of error terms of these items was therefore considered to be appropriate in the present analysis.

### Ethical considerations

The study was approved by the local Ethics Committee, Karolinska Institutet, Dnr. 01-224. All data were handled anonymously. Participation was voluntary, and the patients could withdraw from the study at any time.

## Results

Demographic data and illness-related factors for the 195 patients included in the analyses are presented in Table 1. The descriptive statistics for the four factors are shown in Table 2.

**Table 1:** Descriptive Statistics, Demographic and Illness-related Factors

	N = 195
Diagnosis CD/UC (n)	81/114
Disease duration (mean years/range)	8/1-23
Male/Female (n)	97/98
Age (mean/range)	45/17-75
Spouse/Single (n)	144/51
Education: < 12 years/>12 years	29/166
Smoker/Ex-smoker/Never smoked (n)	34/83/78

**Table 2:** Summary of Statistics for the RFIPC

	Median	Mean	SD	Range
Total sample (n = 195)				
- RFIPC 1 (Impact of disease)*	303	392.3	318.78	0 - 1198
- RFIPC 2 (Sexual intimacy)**	26	68.2	85.70	0 - 300
- RFIPC 3 (Complications)***	140	158.1	123.26	0 - 400
- RFIPC 4 (Body stigma)***	20	48.6	59.94	0 - 200
				
Males (n = 97)				
- RFIPC 1 (Impact of disease)*	232	356.6	317.35	0 - 1198
- RFIPC 2 (Sexual intimacy)**	24	60.4	78.27	0 - 295
- RFIPC 3 (Complications)***	130	145.5	112.03	0 - 400
- RFIPC 4 (Body stigma)****	13	42.4	57.60	0 - 200
				
Females (n = 98)				
- RFIPC 1 (Impact of disease)*	388	427.4	317.90	0 - 1131
- RFIPC 2 (Sexual intimacy)**	34	75.8	92.17	0 - 300
- RFIPC 3 (Complications)***	147	170.4	132.78	0 - 400
- RFIPC 4 (Body stigma)****	29	54.8	61.83	0 - 200
				
Crohn’s Disease (n = 81)				
- RFIPC 1 (Impact of disease)*	443	448.0	324.19	0 - 1198
- RFIPC 2 (Sexual intimacy)**	50	78.0	86.64	0 - 300
- RFIPC 3 (Complications)***	158	174.8	122.43	0 - 400
- RFIPC 4 (Body stigma)****	34	56.6	62.02	0 - 200
				
Ulcerative colitis (n = 114)				
- RFIPC 1 (Impact of disease)*	258	353.3	310.42	0-1186
- RFIPC 2 (Sexual intimacy)**	14	61.3	84.74	0-295
- RFIPC 3 (Complications)***	106	146.4	123.02	0-400
- RFIPC 4 (Body stigma)****	13	43.0	58.06	0-200

*Possible score 0 - 1300, ** Possible score 0 - 300, ***Possible score 0 - 400, ****Possible score 0 - 200

The fit statistics for the CFA models are presented in Table 3. It can be seen that the single-factor model displayed poor fit indices. The significant Chi-square (Chi^2^_209_ = 1227.2; p < 0.0001), together with SRMR (0.074) and RMSEA (0.159) exceeding the critical thresholds, and a low CFI (0.73) indicate inadequate fit of the single-factor model to the data.

**Table 3:** Fit Indices for CFA Models of the RFIPC

Model	X^2^	df	CFI	SRMR	RMSEA
Single-factor model	1227.2	209	0.73	0.074	0.159
Four-factor model	601.8	203	0.827	0.061	0.101
Four-factor model, CE permitted	427.2	201	0.902	0.056	0.076

The four-factor model displayed better fit indices than the single-factor model but failed to approximate the established thresholds for the fit indices [45-46]. The Chi-square (Chi^2^_203_ = 601.8; p < 0.0001) remained significant. However, regarding the Chi-square statistics in both models, it should be noted that Chi-square indices are limited in that they tend to approach significance even in well-fitting models [37]. Further fit indices provide better means of examining the fit of the model. The four-factor model’s SRMR (0.061) and RMSEA (0.101) and CFI (0.827) mark substantive improvement of the model fit over the single factor, but still remain inadequate.

**Figure 1:**
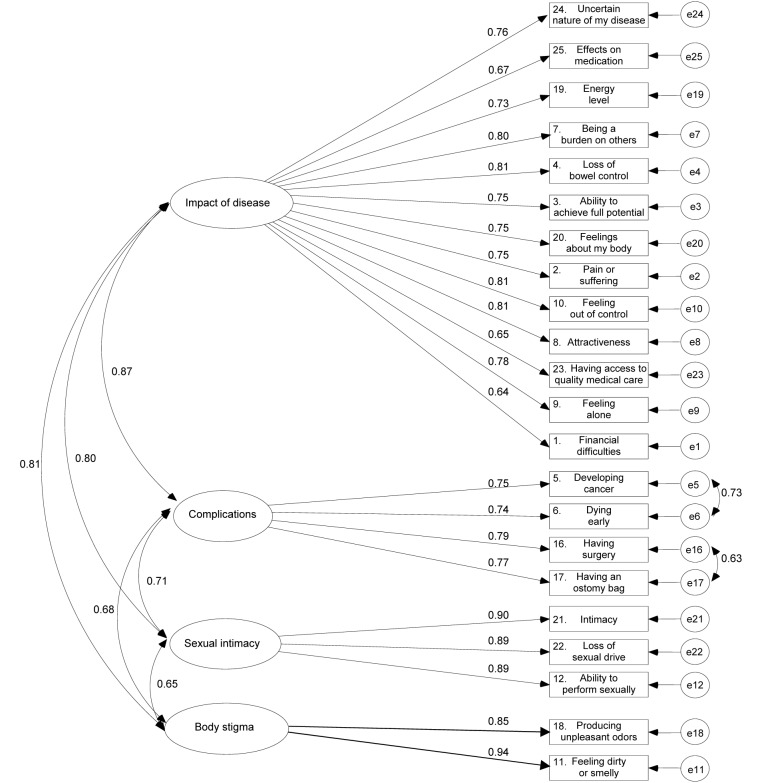
Graphical representation of the correlated four-factor model of the RFIPC. The factor loadings are standardized loadings.

The pre-specified final four-factor model permitting correlated error terms between items 5 and 6, and items 16 and 17 displayed the most adequate fit. The model’s Chi-square remained significant (Chi^2^_201_ = 427.24; p < 0.0001), whereas SRMR (0.056) and RMSEA (0.076) decreased and the CFI (0.902) reached an acceptable level of model fit as suggested by early recommendations [47]. A schematic representation of this factor structure is presented in Figure 1**.**

Cronbach’s alpha coefficients were calculated for each factor according to the final model. For factor 1, impact of disease, Cronbach’s alpha coefficient was 0.94; for factor 2, sexual intimacy, 0.92; for factor 3, complications of the disease, 0.89; and for factor 4, body stigma, 0.88 (n = 195).

Significant correlations were found between the four factors of the RFIPC and the four factors of the IBDQ and the HI (Table 4). The correlation between the four factors of the RFIPC and emotional function showed the highest correlation coefficients, while correlation coefficients regarding systemic symptoms showed the lowest correlations.

**Table 4:** Spearman’s Correlation Coefficient Describing Relationship Between RFIPC Factors and IBDQ Factors and Health Index (HI)

	IBDQ 1	IBDQ 2	IBDQ 3	IBDQ 4	HI
	Bowel symptoms	Systemic symptoms	Social function	Emotional function	
RFIPC 1 (Impact of disease)	0.35*	0.46*	0.34*	0.69*	- 0.52*
RFIPC 2 (Sexual intimacy)	0.31*	0.34*	0.37*	0.55*	- 0.43*
RFIPC 3 (Complications)	0.27	0.32*	0.20	0.62*	- 0.29*
RFIPC 4 (Body stigma)	0.29*	0.29*	0.18	0.53*	- 0.44*

* Significant p-values.

Mann-Whitney U analyses showed that patients with CD and UC scored significantly different on the factor impact of disease. The patients with CD reported greater worries and concerns regarding this factor (U = 3760.5, p = 0.038). There were no significant differences between the diagnoses on the other three factors.

## Discussion

In the present study the factor structure of the Swedish version of the RFIPC was validated. The RFIPC has been widely used as a tool for assessing HRQOL in patients with IBD. At present, there are a number of other measures specifically aimed to evaluate HRQOL in IBD, for example the Inflammatory Bowel Disease Questionnaire (IBDQ), the Short Health Scale (SHS), and the Padova Inflammatory Bowel Disease Quality of Life (PIBDQL) [41, 43, 48-49]. Each of these measures provides unique information relating to the multi-dimensional construct of HRQOL. The impact of psychological factors associated with HRQOL is recognized and items tapping some of these factors are included in several of these measures, such as anger, depression, irritability, worry, and well-being [7-8, 11, 41, 48-49]. However, the RFIPC stands out as a unique instrument in this context by focusing exclusively on the content and degree of patients’ worries and concerns [1-2]. As stated by Drossman et al [1], these worries comprise a main adverse influence on the patient’s health and well-being. Our clinical observations point to confirmation of this suggestion. Consequently, a thorough validation of the RFIPC is essential, as detailed assessments of patients’ worries are not provided by any other HRQOL instruments that are currently accessible. The present study focused on validation of the factorial structure of the Swedish version of the RFIPC since to our knowledge, differentiation of the components of worries, as put forward by Drossman et al [1], has not been addressed in previous studies. The findings in the present study provide support for the structural validity of the Swedish version of the RFIPC.

The present results suggest that a single-factor model of the RFIPC, i.e. considering a single underlying uni-dimensional latent variable of the patient’s worries that is manifested in the items of the RFIPC, is an unacceptable structure for this measure. Approaching the RFIPC as a single-factor measure implies that a patient may be characterized by a degree of worries and concerns, no matter what these worries are about. This single-factor approach also corresponds to a scoring procedure in which the sum of all items of the RFIPC provides an assessment of the patient’s general worries and concerns. This scoring procedure has been used in several studies utilizing the Swedish version of the RFIPC [4-9, 36].

A slightly modified model of the original factorial structure of the RFIPC displayed the most acceptable fit in the present study. According to the results, the RFIPC is best described as a multi-dimensional measure in which patients’ worries and concerns could be divided into distinct components: impact of disease, sexual intimacy, complications, and body stigma. This approach to the RFIPC suggests that a patient, or a subgroup of patients, may have concerns related to complications, while others may be worried about other aspects of living with IBD. These differentiations between the components of worries in patients with IBD not only provide more nuanced data for studying the HRQOL in IBD, but also provide useful information in clinical settings. By recognizing patients’ areas of concern, efforts such as patient education and support may be more directed to individual needs.

The findings in the present study lend further support to this differential pattern of worries, by showing that social function as an aspect of HRQOL in IBD is associated with worries concerning impact of the disease and sexual intimacy, but not with concerns about complications or body stigma. Furthermore, the results from the present study suggest that bowel symptoms are unrelated to worries and concerns about complications. Earlier studies showed that a low perceived level of information had a strong correlation with patients' worries and concerns, and patients with major worries regarding impact of the disease expressed a need for psychosomatic support [24, 31]. In previous studies, higher levels of concerns regarding impact of the disease, complications, sexual intimacy, and body stigma have shown to be associated with increasing disease severity; and major worries and concerns about impact of the disease were associated with female sex [26-27]. The possibility of acquiring more detailed information regarding patients’ worries by the suggested hypothesized four factors structure was further displayed by corroborated by findings of the present study, indicating that patients with CD reported significantly greater worries and concerns in the factor impact of disease in comparison with patients with UC.

Worry is a common human experience with a predominance of verbal thought whose function appears to be the cognitive avoidance of threat [50]. The highest rated reasons for worrying are that it helps individuals discover ways of avoiding negative future events and it prepares them for the worst scenario if they cannot avoid it [51]. People with chronic diseases may have major limitations in physical, emotional, social and occupational functioning. It has been shown that psychological factors, in particular cognitive representations and coping efforts, play a crucial role in adaptation to a chronic disease [52]. Patients with chronic diseases who view their illness as more serious and less controllable have poorer health outcomes [53]. Worry can be an important factor in the development of negative cognitive representations of illness in individuals facing a life-threatening illness [54]. Worry and rumination also correlates positively with disengagement of coping effects, and negatively with perceived coping effectiveness [55].

Worries and concerns about IBD may affect the patient’s adaptation to the illness and may also have an effect on the clinical outcome. Major worries about IBD are associated with a depressive cooping style, characterized by feelings of helplessness, social withdrawal and self-pity [24, 26]. A more detailed assessment of the disease-related worries and concerns of the IBD patients provides useful information about HRQOL and enables identification of the kind of worries and concerns that are most important to each patient [2, 24, 26-28, 31, 34]. The factorial structure established in the present study improves the utility of the Swedish version of the RFIPC by providing assessments of areas that are most important to concentrate on with regard to research, education, and support.

It should be noted that although the obtained CFI in the present study, according to Kline (2005), indicates a good fit for the hypothesized model [56], it falls shortly below the more stringent recommendations for cut-off for superior fit (> 0.95) advised by Hu and Bentler, 1999 [46]. Furthermore, the analysis in the present study is performed in a single primary sample, and thus, is not cross validated. To establish the results of the present study more firmly, and to address these limitations, a cross validation of our suggested factor structure of the RFIPC in other samples is advisable. The results of the present study, however, provide encouraging indications for the adaptation of the original scoring procedure in the Swedish settings.

In conclusion, the factorial structure of the RFIPC as suggested by Drossman et al [1] was able to be replicated, although with a slight modification, in the Swedish version. The separate factors identified in this structure provide more detailed information about the disease-related worries and concerns of IBD patients in both research and clinical settings as these components of worries are different related to HRQOL and general health. These components also may differ in subgroups of patients with IBD. The more detailed assessment provided by this validated structure of the RFIPC may help physicians and nurses in providing each patient the appropriate education and support, by directing the efforts toward the specific areas of worries and concerns that are most prominent.
